# Cost-effectiveness and budget impact of decentralising childhood tuberculosis diagnosis in six high tuberculosis incidence countries: a mathematical modelling study

**DOI:** 10.1016/j.eclinm.2024.102528

**Published:** 2024-03-21

**Authors:** Marc d’Elbée, Martin Harker, Nyashadzaishe Mafirakureva, Mastula Nanfuka, Minh Huyen Ton Nu Nguyet, Jean-Voisin Taguebue, Raoul Moh, Celso Khosa, Ayeshatu Mustapha, Juliet Mwanga-Amumpere, Laurence Borand, Sylvie Kwedi Nolna, Eric Komena, Saniata Cumbe, Jacob Mugisha, Naome Natukunda, Tan Eang Mao, Jérôme Wittwer, Antoine Bénard, Tanguy Bernard, Hojoon Sohn, Maryline Bonnet, Eric Wobudeya, Olivier Marcy, Peter J. Dodd, TB-Speed Health Economics Study Group, Doris Arlt-Hilares, Eric Balestre, Marie-France Banga, Antoine Bénard, Tanguy Bernard, Maryline Bonnet, Laurence Borand, Guillaume Breton, Dim Bunnet, Paul-Damien Chateau, Saniata Cumbe, Marc d’Elbée, Agathe de Lauzanne, Peter James Dodd, Martin Harker, Minh Huyen Ton Nu Nguyet, Sanary Kaing, Celso Khosa, Eric Komena, Monica Koroma, Sylvie Kwedi Nolna, Nyashadzaishe Mafirakureva, Tan Eang Mao, Olivier Marcy, Douglas Mbang Masson, Raoul Moh, Jacob Mugisha, Ayeshatu Mustapha, Juliet Mwanga-Amumpere, Mastula Nanfuka, Naome Natukunda, Joanna Orne-Gliemann, Eric Ouattara, Julien Poublan, Hojoon Sohn, Jean-Voisin Taguebue, Immaculate Tulinawe, Yara Voss de Lima, Jérôme Wittwer, Eric Wobudeya

**Affiliations:** a University of Bordeaux, National Institute for Health and Medical Research (Inserm) UMR 1219, Research Institute for Sustainable Development (IRD) EMR 271, Bordeaux, France; b Ceped UMR 196, Université Paris Cité, Research Institute for Sustainable Development (IRD), Inserm, Paris, France; c TB Modelling Group, TB Centre, and Global Centre for Health Economics, London School of Hygiene and Tropical Medicine, London, United Kingdom; d School of Health & Related Research, University of Sheffield, Sheffield, United Kingdom; e MU-JHU Care Ltd, MUJHU Research Collaboration, Kampala, Uganda; f Mother and Child Center, Chantal Biya Foundation, Yaoundé, Cameroon; g Teaching Unit of Dermatology and Infectiology, UFR of Medical Sciences, Félix-Houphouët Boigny University, Abidjan, Côte d’Ivoire; h Programme PAC-CI, CHU de Treichville, Abidjan, Côte d’Ivoire; i Instituto Nacional de Saúde, Marracuene, Mozambique; j Ola During Children's Hospital, Freetown, Sierra Leone; k Epicentre Mbarara Research Centre, Mbarara, Uganda; l Epidemiology and Public Health Unit, Clinical Research Group, Institut Pasteur du Cambodge, Phnom Penh, Cambodia; m Center for Tuberculosis Research, Division of Infectious Diseases, Johns Hopkins University School of Medicine, Baltimore, MD, USA; n TransVIHMI, University of Montpellier, IRD /INSERM, Montpellier, France; o SOLTHIS, Sierra Leone; p Ministry of Health, Cambodia; q University of Bordeaux, National Institute for Health and Medical Research UMR 1219, Bordeaux, France; r CHU Bordeaux, Service d'information Médicale, USMR & CIC-EC 14-01, Bordeaux, France; s Bordeaux School of Economics, Bordeaux, France; t Seoul National University College of Medicine, Seoul, South Korea

**Keywords:** Paediatric tuberculosis, Decentralisation, Diagnosis, Economic evaluation, Low- and middle-income countries

## Abstract

**Background:**

The burden of childhood tuberculosis remains high globally, largely due to under-diagnosis. Decentralising childhood tuberculosis diagnosis services to lower health system levels could improve case detection, but there is little empirically based evidence on cost-effectiveness or budget impact.

**Methods:**

In this mathematical modelling study, we assessed the cost-effectiveness and budget impact of decentralising a comprehensive diagnosis package for childhood tuberculosis to district hospitals (DH-focused) or primary health centres (PHC-focused) compared to standard of care (SOC). The project was conducted in Cambodia, Cameroon, Côte d’Ivoire, Mozambique, Sierra Leone, and Uganda between August 1st, 2018 and September 30th, 2021. A mathematical model was developed to assess the health and economic outcomes of the intervention from a health system perspective. Estimated outcomes were tuberculosis cases, deaths, disability-adjusted life years (DALYs) and incremental cost-effectiveness ratios (ICERs). We also calculated the budget impact of nationwide implementation. The TB-Speed Decentralization study is registered with ClinicalTrials.gov, NCT04038632.

**Findings:**

For the DH-focused strategy versus SOC, ICERs ranged between $263 (Cambodia) and $342 (Côte d’Ivoire) per DALY averted. For the PHC-focused strategy versus SOC, ICERs ranged between $477 (Cambodia) and $599 (Côte d’Ivoire) per DALY averted. Results were sensitive to TB prevalence and the discount rate used. The additional costs of implementing the DH-focused strategy ranged between $12.8 M (range 10.8–16.4) (Cambodia) and $50.4 M (36.5–74.4) (Mozambique), and between $13.9 M (12.6–15.6) (Sierra Leone) and $134.6 M (127.1–143.0) (Uganda) for the PHC-focused strategy.

**Interpretation:**

The DH-focused strategy may be cost-effective in some countries, depending on the cost-effectiveness threshold used for policy making. Either intervention would require substantial early investment.

**Funding:**

Unitaid.

**Research in context**

**Evidence before this study**

We searched the PubMed database using (tuberculosis) AND ((paediatric) OR (pediatric) OR (child∗)) AND ((costs and cost analysis) OR (“budget impact analysis”)) between January 1st, 2000 and November 30th, 2023, without language restrictions. We found 19 articles assessing the cost-effectiveness of a broad range of interventions for child tuberculosis such as intensified case finding and strengthened household contact management, and no budget impact analysis of these interventions. A study published by Thompson et al., in 2023 investigated the costs and cost-effectiveness of a decentralised molecular testing strategy for adult tuberculosis in Uganda, and recommended decentralised Xpert testing. In 2022, WHO recommended decentralised models of care to deliver tuberculosis services to children but rated the overall certainty of evidence as “very low”, with no evidence on costs and cost-effectiveness. To date, the TB-Speed Decentralization study is the closest assessment of the intervention recommended by the WHO.

**Added value of this study**

Unlike all of the published studies, our intervention investigated a decentralised comprehensive package of care for children as developed by the TB-Speed project which includes systematic tuberculosis screening for all sick children <15 years, clinical evaluation, Xpert MTB/RIF Ultra-testing on respiratory and stool samples, and chest radiography for children with presumptive tuberculosis. This study found that, compared to the standard of care, decentralising a comprehensive diagnosis package for childhood tuberculosis to district hospitals is potentially cost-effective from a health systems perspective while decentralising to primary health centres is unlikely to be cost-effective. Decentralisation would require substantial financial investment in the early implementation phase for equipment purchases.

**Implications of all the available evidence**

Decentralisation of tuberculosis diagnostic services could be cost-effective in some settings with high prevalence of tuberculosis in children seeking healthcare. The main factors affecting cost-effectiveness are the level of decentralisation (district hospital versus primary health centre), local tuberculosis prevalence, and facility testing volumes. Substantial financial commitment is needed in the early implementation phase. Following WHO recommendations, countries should consider scaling up locally-adapted interventions to improve diagnosis of tuberculosis and other diseases using Xpert machines to improve cost-effectiveness, prioritising areas with highest tuberculosis prevalence, and include such plans when identifying domestic or donor sources of funding.

## Introduction

Tuberculosis mortality remains high in children globally,^1^ with 209,000 deaths estimated by the World Health Organization (WHO) for 1.1 million paediatric cases in 2021.^2^ Modelling suggests the majority (96%) of these deaths are occurring among children not receiving treatment for tuberculosis.^3^ In 2021, only 38.5% of childhood tuberculosis cases were reported to the WHO, largely because of substantial underdiagnosis which prevents children from receiving treatment.

Diagnosing tuberculosis in children is challenging largely due to difficulties in collecting expectorated sputum samples and insufficient bacteria in samples to test positive due to the paucibacillary nature of pulmonary tuberculosis in children.^4^, ^5^, ^6^ Alternative specimen collection methods such as induced sputum and gastric aspirate require equipment and experienced personnel, which are often lacking at primary health centre (PHC) level where most sick children seek care, and may be unavailable at district hospital (DH) level. Consequently, most children with presumptive tuberculosis (symptoms suggestive of tuberculosis) do not access appropriate diagnostic tests for tuberculosis, even when seen at DH level.^7^ Referrals introduce potential delays, risk losses to follow-up, and do not align with the ambition of providing patient-centred tuberculosis care.^8^

Developments in molecular diagnostics and novel sample collection procedures provide opportunities to decentralise diagnostic capacity for paediatric tuberculosis to the primary care level. While more costly than smear microscopy, the higher sensitivity, robustness and low training requirements of the WHO-endorsed GeneXpert-operated rapid molecular diagnostic assays for tuberculosis^9^ allow their deployment at PHC level. Stool samples can be collected in young children regardless of setting or equipment compared to respiratory samples and can be used to identify *Mycobacterium tuberculosis* using Xpert MTB/RIF.^10^^,^^11^ Nasopharyngeal aspirates (NPA) are easier to collect than gastric aspirate or induced sputum sampling, and in combination with stool samples can provide similar sensitivity to Xpert MTB/RIF testing on two gastric aspirates or induced sputa.^10^ The WHO has now recommended these sample collection methods with Xpert MTB/RIF Ultra, the latest generation of molecular tests, for paediatric tuberculosis diagnosis.^9^

The recent revision to the WHO guidelines for child and adolescent tuberculosis recommended decentralised models of care to notably increase case detection in children.^9^ However, this recommendation was provisional due to the low quality of evidence, including evidence on cost-effectiveness, for such approaches. Few published studies evaluate models of care for paediatric tuberculosis, and even fewer include economic evaluation; none to our knowledge has included a budget impact analysis. For many countries with limited resources and high tuberculosis incidence, objectively weighing trade-offs between policy options and considering their affordability is crucial, and is explicitly required when applying for donor support including Global Fund.

The TB-Speed Decentralization study (NCT04038632) used a pre−/post-intervention approach to assess the impact on tuberculosis case detection of decentralising a comprehensive childhood tuberculosis diagnosis package at DH or PHC level.^12^ The package included systematic outpatient screening, Xpert MTB/RIF Ultra on stool and NPA samples for those with presumptive tuberculosis, enhanced healthcare worker training and clinical mentoring to improve clinical skills, and digital chest X-ray at DH level. The study was conducted in 12 DHs and 47 PHCs across six high tuberculosis incidence countries: Cambodia, Cameroon, Côte d’Ivoire, Mozambique, Sierra Leone, and Uganda. In each country, two rural or semi-urban districts were randomly allocated to either a PHC-focused or DH-focused decentralisation strategy. In the DH-focused strategy, children with presumptive tuberculosis at PHC level were referred to DH for diagnosis; in the PHC-focused strategy, diagnostic evaluations were performed at PHC, except chest X-ray done at DH if required. Baseline ‘pre’ data comprised 9 months of retrospective data and at least 3 months of prospective data. Comparator ‘post’ data were collected for 12 months after introducing the intervention. This study found that implementing systematic screening for tuberculosis among sick children under 15 years attending care, and a full clinical evaluation, Xpert Ultra testing of NPA and stool or expectorated sputum, and chest X-Ray using a standardised approach in those identified with presumptive tuberculosis nearly tripled childhood tuberculosis case detection as compared to pre-intervention data. The DH-focused approach had a larger effect on childhood tuberculosis case detection than the PHC-focused approach.

In this analysis we sought to fill the evidence gaps on the economic evaluation of real-world strategies for improving paediatric tuberculosis care by undertaking a cost-effectiveness and budget impact analysis of the TB-Speed Decentralization study.^12^ We used empirical data on costs and the cascades of tuberculosis care (screening, clinical assessment, microbiological/radiological testing, and treatment) from the study with mathematical models of patient pathways to compare the health and cost consequences of the different models of care.

## Methods

### Patient pathways

**Fig. 1:**
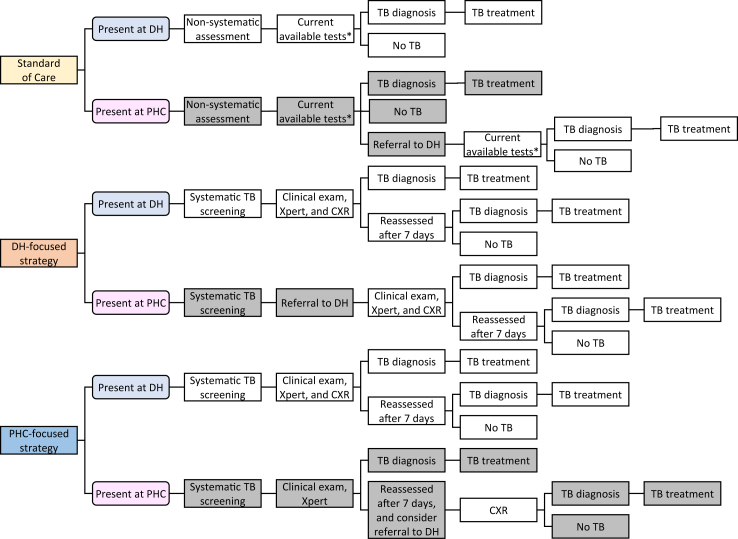
Simplified patient care pathways for the diagnosis and treatment of tuberculosis in children ∗Clinical exam, and, for a proportion of patients, Xpert on sputum or gastric aspirate, smear microscopy and CXR (in DH). “Xpert” in the DH-focused and PHC-focused strategies means “Xpert Ultra on NPA & stool”. We define “non-systematic assessment” as a step in the patient care pathway where a child presenting to the health facility may receive consideration for the possibility of having TB, depending on the clinician and patient. This screening may contain some of the components of our systematic screening and/or some form of clinical examination, but it would not be expected to follow the precise protocols that define the “systematic screening” and “clinical examination” in our intervention arms. TB, tuberculosis; DH, district hospital; PHC, primary health centre; CXR, chest X-ray.

Conceptual models were developed through iterative consultation with country experts and the TB-Speed Decentralization study team to represent detailed patient care pathways for three comparator arms: a standard of care (SOC), the DH-focused strategy and the PHC-focused strategy (Fig. 1 and Appendix section II). Diagrammatic representations of patient pathways formed the basis of the decision-analytic model structure; accompanying narrative descriptions of activities at each stage informed quantification of resource use. We sought to develop pathways that were general enough to include common elements across all included countries. The SOC pathway represented expert consensus on typical care available in high tuberculosis incidence countries, informed by the baseline facility assessment from the included countries before the start of the TB-Speed Decentralization intervention. DH-focused and PHC-focused pathways reflected the TB-Speed Decentralization protocol, to represent the expected patient pathway if these interventions were implemented widely across the target countries.

### Costing approach

#### Cost data collection

Cost data collection tools were adapted from the Value TB costing tool suite developed in conjunction with the Global Health Cost Consortium (GHCC), with reference to the GHCC/WHO guidance ‘Costing Guidelines for Tuberculosis Interventions’.^13^

Labour costs were sourced from national pay scales and project accounts, medications from the Stop TB partnership Global Drug Facility catalogue,^14^ consumables including materials for diagnostic tests, staff training, and equipment from project accounts, and hospitalisation cost of an inpatient bed day from the WHO-CHOICE unit cost estimates for service delivery.^15^ As this intervention did not change facility infrastructure, we excluded facility-associated overhead costs from the analysis. More information on the scope of the costing can be found in Appendix section III.

Routine health facility-level aggregated data on patient numbers were collected by field research assistants from outpatient, tuberculosis, and laboratory registers during the observation (August 1st, 2018–November 30th, 2019), preparatory and intervention (March 1st, 2020–September 30th, 2021) phases.

To calculate the proportionate use of major equipment such as X-ray or GeneXpert machines we used the expected lifespan and annual number of uses of each item. These data were obtained through key informants at MSF-Logistique (https://www.msflogistique.org/) for expected lifespan and from laboratory managers for number of uses over the course of 12 months at PHCs and at DHs.

#### Time and motion study

To value the contribution of labour we conducted a time and motion study to estimate the length of time that staff spent on each patient care task under TB-Speed interventions. Timesheets were used to record the length of tuberculosis-related consultations, appointments and diagnostic tests. Healthcare workers were invited to take part voluntarily and no personal data were collected, only the site name and the role of the healthcare worker. All participants signed an informed consent form. On the self-completed timesheets, participants recorded all activities related to TB-Speed patients and the length of their time they spent on each activity. Participants included doctors, clinical officers, nurses, laboratory technicians and radiographers, at DH and PHC levels in all countries, with 179 participants in total.

#### Cost analysis

Unit costs were estimated using an ingredient-based costing approach, in which the expected resources required for each child enrolled into the study were listed, costed, and summed to estimate direct health service utilisation and cost varying by patient characteristics and the route taken on the pathway. Services (number and type of diagnostic and treatment procedures, materials, laboratory investigations and medications) provided to patients were valued by multiplying the quantities required by their unit costs. The value of all time spent by staff for each patient was estimated as the product of ‘hours spent’ and ‘hourly labour costs’. Costs were estimated in 2021 US dollars (USD), using a discount rate of 3% for the annualisation of the economic costs of equipment following guidelines.^16^^,^^17^

### Modelling approach

A decision analytic mathematical model was developed in R software to assess the clinical benefits, cost-effectiveness, and budget impact of the intervention from a health system perspective. The attributes of children flowing through the tree were: age (0–4 years or 5–14 years); HIV and antiretroviral treatment status (each positive or negative); and true tuberculosis status (bacteriologically-confirmed tuberculosis, bacteriologically-unconfirmed tuberculosis, not tuberculosis). The attribute ‘bacteriologically confirmed tuberculosis’ refers to tuberculosis that would be bacteriologically positive under ideal circumstances and with all samples available.

The probabilities of following each route through the tree depended on attributes, and were parameterized using a mixture of literature, TB-Speed Decentralization study data, and expert opinion. Literature was used to parametrize the accuracy of diagnostic algorithms and availability of samples. Study data on child characteristics, level of presentation, and the care cascade were used to calibrate tuberculosis prevalence, the level of initial care seeking (given true tuberculosis status), and the likelihoods of being assessed and being considered to have presumptive tuberculosis. Expert opinion was used to inform unobserved features of typical care such as referral loss to follow-up and the frequency with which certain diagnostic tests were used under standard of care. Modelling of the cascade of care and intervention effect was based on calibration of unobserved parameters to meta-analytic summaries, representing a typical cascade. We undertook sensitivity analyses exploring the influence of tuberculosis prevalence, assumed constant across countries in the model, and of the discount rate applied. See Appendix section IV for more details.

Country-specific unit costs associated with resource use at each step of care were accumulated to produce total mean costs. Health benefits for those with tuberculosis were modelled with a previously published approach that used case-fatality ratio from systematic literature reviews to quantify mortality reductions from treating more tuberculosis.^3^ Country-specific life expectancy from United Nations estimates was used to calculate the mean life-years lost over a lifetime horizon (with and without 3% discounting). We disregarded the contribution of morbidity to disability adjusted life-years (DALYs). All results were calculated using a probabilistic sensitivity analysis with 1000 replicates.

We report the total and incremental (to SOC) number of children treated for tuberculosis, costs, number of deaths and deaths averted, number of DALYs and DALYs averted, per 100,000 children presenting as outpatients, and the incremental cost-effectiveness ratios (ICERs) for both interventions in each country. ICERs were compared to various options for cost-effectiveness thresholds (presented as a range) in each country to assess potential cost-effectiveness.^18^^,^^19^ We complied with the Consolidated Health Economic Evaluation Reporting Standards (CHEERS 2022) reporting guidelines.^20^

To project the 5-year (2022–2026) budget impact of adopting these interventions nationally in each country, we fitted a cost function in Excel using parameters related to costs of equipment and its delivery, installation and maintenance, training, supplies, and personnel costs.^21^ Input quantities were the national number of DHs, PHCs, eligible healthcare workers, and pre-intervention (2019) annual rate of tuberculosis notification in children 0–14 years old from the WHO Global TB Programme. See Appendix section V for additional details. Costs were combined with the scale of deployment to generate the required implementation budget in each year for each country. We assumed a 3-year period (2022–2024) to provide facilities with equipment, to train all eligible staff, and rollout supply chain delivery to all sites. A sensitivity analysis is presented in Appendix section V.

A health economic analysis plan was developed jointly by economists, modellers, international and country investigators. The economic analyses were nested within the main study protocol approved by the WHO Ethical Review Committee, Inserm's ethics review committee (IRB00003888), as well as all national ethics committees prior to the start of the study (See Appendix section VI).

### Role of the funding source

The study funders had no role in the study design, data collection, data analysis, data interpretation, or writing of the report. The corresponding author had full access to all the data in the study and had final responsibility for the decision to submit for publication.

## Results

**Fig. 2:**
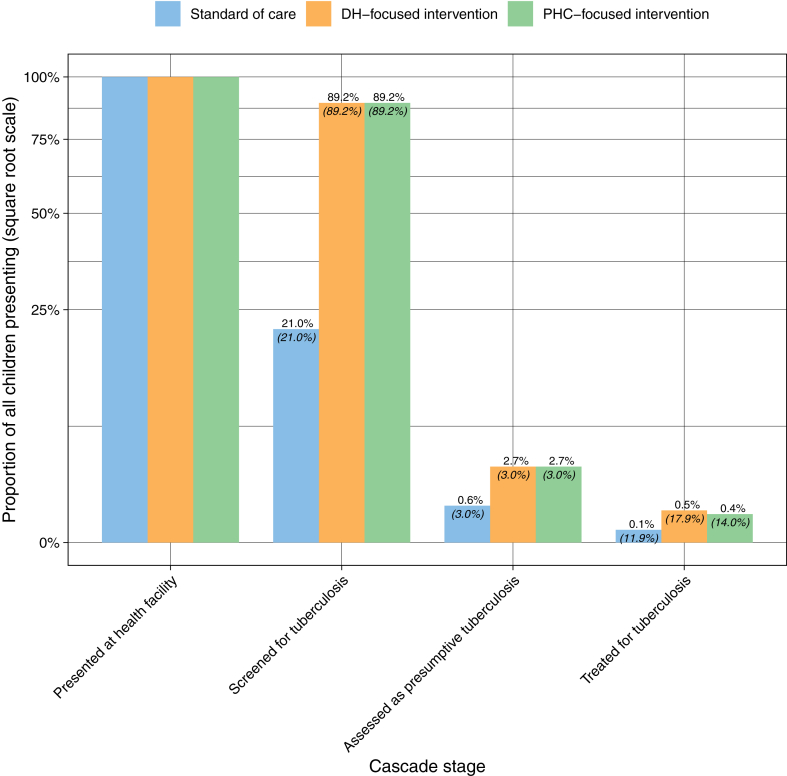
Screening to diagnosis cascade during the intervention period by decentralisation approach Percentages are bar height both with respect to the previous step (italic parentheses below bar), and also overall (normal font atop the bar).

Overall, the modelled cascade of care data showed that the tuberculosis screening rate among children presenting to health facilities increased from 21% (SOC) to 89% (DH-focused and PHC-focused), the rate of children identified with presumptive tuberculosis among screened children remained constant (3%), and the rate of children initiated on treatment among the children identified with presumptive tuberculosis increased from 12% (SOC) to 18% (DH-focused) and 14% (PHC-focused) (Fig. 2). Results were similar between 0–4 and 5–14 years age groups (Appendix section IV).

Results from the cost analysis and the time and motion study, as well as costs per child treated disaggregated by arm, cost category, and tuberculosis care stage are presented in the Appendix section III. Compared to 74 (95% uncertainty interval [UI]: 6–257) children treated for tuberculosis in the SOC (per 100,000 presenting), we estimated 476 (95% UI: 104–1210) treated in the DH-focused arm and 373 (95% UI: 80–925) treated in the PHC-focused arm (Table 1). Compared with the SOC at 44 (6–131) deaths per 100,000 presenting, the number of deaths averted was 21 (2–69) and 18 (2–57), for the DH-focused and PHC-focused interventions, respectively. For the DH-focused intervention costs per 100,000 increased by between $158 K ($41–357 K) in Cambodia and $190 K ($54–416 K) in Côte d’Ivoire, while DALYs averted ranged from 547 (54–1754) in Sierra Leone to 600 (59–1916) in Cambodia.

**Table 1:** Health impact, costs and cost-effectiveness.

Arm	Type of outcome	Per 100,000 OPD initial attendances	Cambodia	Cameroon	Côte d’Ivoire	Mozambique	Sierra Leone	Uganda
SOC	Total	Children treated for TB^a^ (95% UI)	74 (6–257)	74 (6–257)	74 (6–257)	74 (6–257)	74 (6–257)	74 (6–257)
		Deaths (95% UI)	44 (6–131)	44 (6–131)	44 (6–131)	44 (6–131)	44 (6–131)	44 (6–131)
		Costs (95% UI)	10,449 (1164–32,054)	13,430 (1453–41,195)	15,574 (1797–46,247)	10,359 (1073–33,665)	10,164 (1100–32,066)	13,581 (1575–40,263)
		DALYs^b^ (95% UI)	1235 (177–3680)	1162 (166–3461)	1145 (163–3410)	1192 (170–3553)	1126 (160–3354)	1200 (171–3576)
DH-focused	Total	Children treated for TB^a^ (95% UI)	476 (104–1210)	476 (104–1210)	476 (104–1210)	476 (104–1210)	476 (104–1210)	476 (104–1210)
		Deaths (95% UI)	23 (3–75)	23 (3–75)	23 (3–75)	23 (3–75)	23 (3–75)	23 (3–75)
		Costs (95% UI)	168,490 (48,731–375,050)	198,455 (57,910–435,159)	205,544 (64,514–440,572)	181,355 (49,499–409,446)	176,682 (46,093–398,673)	193,742 (59,315–415,612)
		DALYs^b^ (95% UI)	635 (87–2100)	597 (82–1971)	588 (81–1943)	613 (84–2025)	579 (79–1909)	617 (84–2039)
	Incremental to SOC	Children treated for TB^a^ (95% UI)	402 (71–1032)	402 (71–1032)	402 (71–1032)	402 (71–1032)	402 (71–1032)	402 (71–1032)
		Deaths averted (95% UI)	21 (2–69)	21 (2–69)	21 (2–69)	21 (2–69)	21 (2–69)	21 (2–69)
		Costs (95% UI)	158,040 (40,967–357,875)	185,025 (50,236–415,776)	189,970 (53,578–415,937)	170,996 (41,951–389,530)	166,518 (38,986–376,362)	180,161 (50,988–395,362)
		DALYs^b^ averted (95% UI)	600 (59–1916)	565 (56–1809)	556 (55–1780)	579 (57–1853)	547 (54–1754)	583 (58–1865)
		ICER^c^	263	328	342	295	304	309
PHC-focused	Total	Children treated for TB^a^ (95% UI)	373 (80–925)	373 (80–925)	373 (80–925)	373 (80–925)	373 (80–925)	373 (80–925)
		Deaths (95% UI)	26 (4–79)	26 (4–79)	26 (4–79)	26 (4–79)	26 (4–79)	26 (4–79)
		Costs (95% UI)	257,022 (71,606–583,728)	295,105 (84,794–661,929)	303,124 (92,655–677,697)	276,615 (73,237–639,476)	273,622 (72,754–628,440)	286,493 (82,536–644,522)
		DALYs^b^ (95% UI)	717 (102–2212)	675 (97–2077)	665 (95–2047)	693 (99–2133)	654 (94–2012)	697 (100–2147)
	Incremental to SOC	Children treated for TB^a^ (95% UI)	298 (52–760)	298 (52–760)	298 (52–760)	298 (52–760)	298 (52–760)	298 (52–760)
		Deaths averted (95% UI)	18 (2–57)	18 (2–57)	18 (2–57)	18 (2–57)	18 (2–57)	18 (2–57)
		Costs (95% UI)	246,572 (64,623–564,187)	281,675 (75,305–640,442)	287,550 (81,529–637,224)	266,256 (66,963–617,904)	263,458 (69,220–612,906)	272,912 (75,041–616,087)
		DALYs^b^ averted (95% UI)	517 (52–1608)	487 (49–1516)	480 (48–1493)	500 (50–1554)	472 (47–1470)	503 (50–1564)
		ICER^c^	477	578	599	533	558	543

SOC, standard of care; TB, tuberculosis; OPD, patients presenting at outpatient department; DH, district hospital; PHC, primary health centre; DALY, disability-adjusted life year; ICER, incremental cost-effectiveness ratio; UI, uncertainty interval.

a We used an average intervention impact, so the number of children treated for tuberculosis by intervention is the same across countries. Costs and life expectancy tables for estimating DALYs are specific to each country.

b Discounted.

c The discrepancy observed between manually calculated and presented ICERs is due to rounding error.

The ICERs compared with the SOC ranged between $263 (Cambodia) and $342 (Côte d’Ivoire) per DALY averted for the DH-focused strategy, and between $477 (Cambodia) and $599 (Côte d’Ivoire) per DALY averted for the PHC-focused strategy. The DH-focused strategy dominated the PHC-focused strategy, being less expensive and more effective. Sensitivity analysis showed that both paediatric tuberculosis prevalence and discount rate applied on life years had a strong impact on the estimated ICERs (Appendix section IV). At a constant TB prevalence of 200/100,000 across age groups, applying no discount rate (0% instead of 3%) led to ICERs decreasing by 57%–60% across countries and strategies. Increasing TB prevalence from 200/100,000 to 500/100,000 (with fixed 3% discounting) led to ICERs decreasing by 55%–56%.

**Fig. 3:**
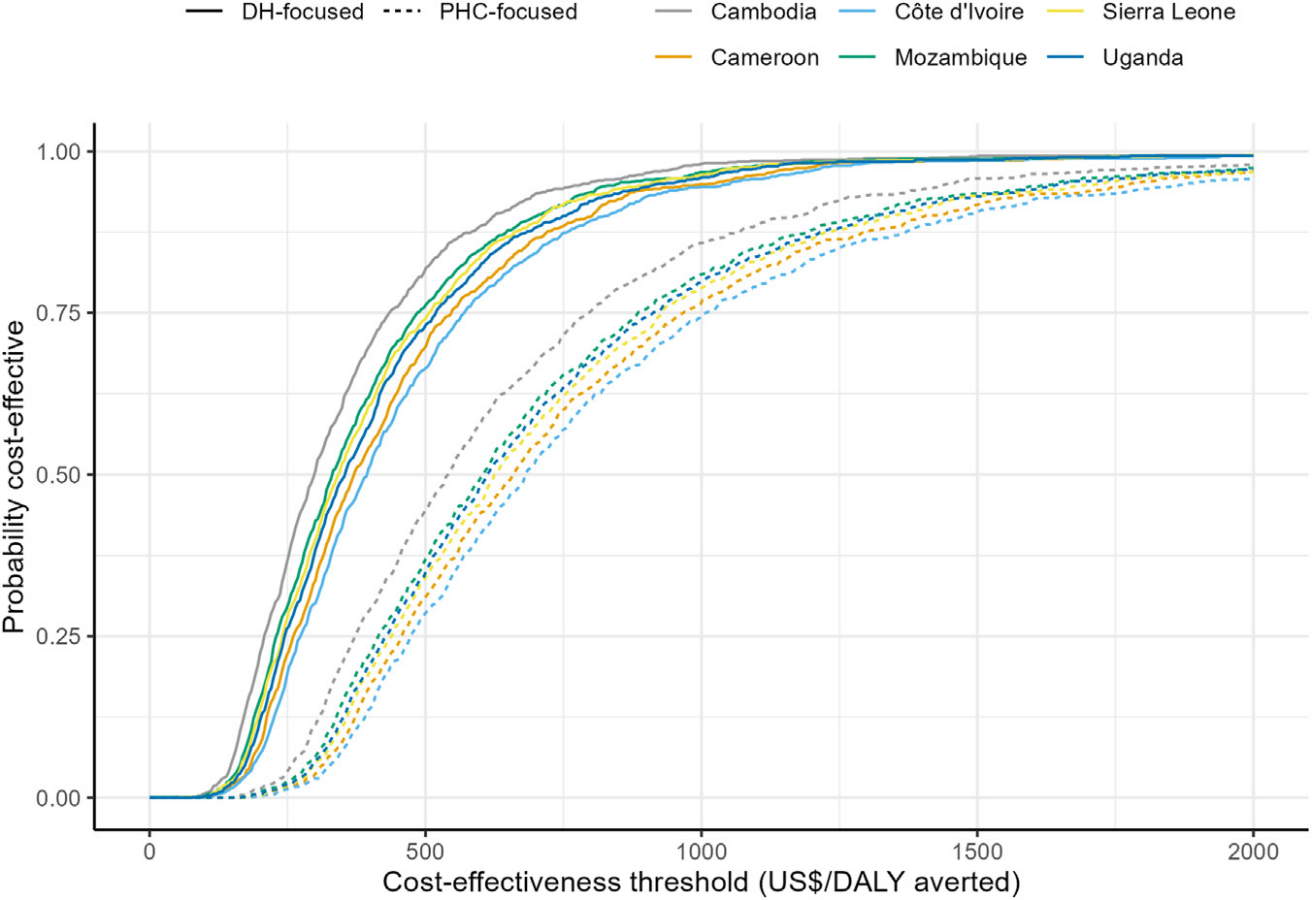
Cost-effectiveness acceptability curves by country for the district hospital-focused and primary health centre-focused strategies, each compared to the standard of care (in US$ per DALY averted). DALY, disability-adjusted life year.

The cost-effectiveness acceptability curves (Fig. 3 and Appendix section IV) show the decision uncertainty surrounding the adoption of the strategies, depending on the cost-effectiveness thresholds which decision-makers may select in each country. Using estimated thresholds (Appendix section IV) from Ochalek et al.,^18^ the highest estimated probabilities of the DH-focused strategy being cost-effective compared to SOC were for Cambodia (40%–69%) and Côte d’Ivoire (49%–65%) and the lowest for Sierra Leone (0%–1%). For the PHC-focused strategy versus SOC, the highest probabilities were for Cambodia (7%–27%) and Côte d’Ivoire (11%–26%) and the lowest for Cameroon, Sierra Leone and Uganda (<1%).

Additional costs of implementing the decentralisation intervention at DH level over five years were estimated between $13 M in Cambodia and Sierra Leone and $50 M in Mozambique, whereas decentralising to PHC level would cost between $14 M in Sierra Leone and $135 M in Uganda (Table 2). Countries with higher numbers of health facilities to equip (Cambodia, Mozambique, Sierra Leone) and/or higher tuberculosis notification rates (Cambodia, Mozambique, Sierra Leone, Uganda) had higher implementation costs. For example, over the 5-year period, for the DH-focused strategy, costs in Mozambique increased from 12% (as a percentage of total costs over the 5-year period) in 2022 to 27% in 2026 because this country has a relatively low number of district hospitals to equip in the first 3 years (2022–2024), and so lower equipment costs incurred, but a relatively high expected number of children to treat (given WHO TB notifications) once decentralisation is fully operational, incurring higher personnel and supplies costs.

**Table 2:** Projected budget impact of adopting the decentralisation intervention by strategy and by country for years 2022–2026.

	Cambodia	Cameroon	Côte d’Ivoire	Mozambique	Sierra Leone	Uganda
**Country indicators in 2021**						
Population (0–14 years), 2021	5,253,000	11,434,000	11,092,000	14,152,000	3,257,000	21,677,000
Annual number of OPD attendances by children 0–14 years, 2020	2,177,000	1,478,000	1,199,000	15,138,000	2,767,000	9,676,000
Annual number of tuberculosis notification for children 0–14 years, 2020	1849	1255	1018	12,856	2350	8218
District hospitals–DH (National)	68	165	150	53	49	163
Primary Health Centres–PHC (National)	1141	2214	3411	1566	242	5155
Ratio PHC/DH	17	13	23	30	5	32
National tuberculosis programme budget, 2021 (millions US$)	34	8	46	30	10	45
**Impact: number of 0–14 year old children treated for tuberculosis over the 5-year implementation period**						
Standard of care	9245	6275	5090	64,280	11,750	41,090
DH-focused (% increase from the SOC)	27,217 (+194%)	18,474 (+194%)	14,985 (+194%)	189,240 (+194%)	34,592 (+194%)	120,969 (+194%)
PHC-focused (% increase from the SOC)	11,467 (+24%)	7783 (+24%)	6313 (+24%)	79,728 (+24%)	14,574 (+24%)	50,965 (+24%)

Budget requirement related to the decentralisation interventions (incremental costs)													
Intervention	Period	Costs (millions US$)	% total costs	Costs (millions US$)	% total costs	Costs (millions US$)	% total costs	Costs (millions US$)	% total costs	Costs (millions US$)	% total costs	Costs (millions US$)	% total costs
DH-focused	2022	2.8 (2.6–3.1)	22%	6.1 (5.9–6.3)	28%	5.5 (5.3–5.8)	28%	6.1 (4.9–7.9)	12%	2.4 (2.2–2.8)	19%	8.7 (7.8–9.9)	18%
	2023	3.2 (2.9–3.7)	25%	6.3 (6––6.8)	29%	5.8 (5.5–6.2)	30%	8.5 (6.5–11.8)	17%	2.8 (2.5–3.5)	22%	10.5 (8.9–13.0)	21%
	2024	3.5 (3.1–4.2)	27%	6.6 (6.2–7.3)	30%	6.0 (5.6–6.6)	31%	10.8 (8.0–15.7)	21%	3.2 (2.7–4.1)	25%	12.2 (10.0–16.1)	25%
	2025	1.5 (1.0–2.4)	12%	1.2 (0.8–2.0)	6%	1.0 (0.7–1.7)	5%	11.3 (7.8–17.6)	22%	2.0 (1.4–3.1)	16%	7.9 (5.2–13.1)	16%
	2026	1.8 (1.2–3.0)	14%	1.5 (1.0–2.5)	7%	1.3 (0.8–2.2)	7%	13.7 (9.4–21.5)	27%	2.4 (1.6–3.8)	19%	9.6 (6.3–16.2)	20%
	Total 2022–2026	12.8 (10.8–16.4)	100%	21.7 (19.9–24.9)	100%	19.6 (18.0–22.5)	100%	50.4 (36.5–74.4)	100%	12.9 (10.4–17.2)	100%	49.0 (38.2–68.5)	100%
PHC-focused	2022	10.0 (9.6–10.4)	32%	21.4 (20.8–22.0)	33%	29.9 (29.1–30.7)	33%	16.1 (14.7–17.7)	27%	4.0 (3.8–4.3)	29%	42.4 (40.7–44.1)	32%
	2023	10.0 (9.6–10.5)	32%	21.5 (20.8–22.1)	33%	30.0 (29.2–30.8)	33%	16.5 (15.0–18.3)	28%	4.1 (3.8–4.4)	29%	42.7 (40.9–44.6)	32%
	2024	10.1 (9.7–10.6)	32%	21.5 (20.9–22.2)	33%	30.0 (29.2–30.9)	33%	16.9 (15.2–19.0)	28%	4.2 (3.9–4.5)	30%	43.0 (41.1–45.1)	32%
	2025	0.6 (0.4–0.9)	2%	0.5 (0.3–0.7)	1%	0.4 (0.3–0.6)	0%	4.7 (3.2–6.7)	8%	0.8 (0.5–1.1)	6%	3.1 (2.1–4.4)	2%
	2026	0.7 (0.4–1.0)	2%	0.5 (0.4–0.8)	1%	0.4 (0.3–0.6)	0%	5.1 (3.5–7.3)	9%	0.8 (0.6–1.2)	6%	3.4 (2.3–4.9)	3%
	Total 2022–2026	31.4 (29.7–33.2)	100%	65.4 (63.2–67.7)	100%	90.7 (88.0–93.6)	100%	59.3 (51.6–69.0)	100%	13.9 (12.6–15.6)	100%	134.6 (127.1–143.0)	100%
**Intervention**	**Average annual costs**	**Costs (millions US$)**	**% of NTP budget**	**Costs (millions US$)**	**% of NTP budget**	**Costs (millions US$)**	**% of NTP budget**	**Costs (millions US$)**	**% of NTP budget**	**Costs (millions US$)**	**% of NTP budget**	**Costs (millions US$)**	**% of NTP budget**
DH-focused	Programme implementation costs (average of 2022–2026)	2.6 (2.2–3.3)	8%	4.3 (4.0–5.0)	54%	3.9 (3.6–4.5)	9%	10.1 (7.3–14.9)	34%	2.6 (2.1–3.4)	26%	9.8 (7.6–13.7)	22%
	Routine costs (2026)	1.8 (1.2–3.0)	5%	1.5 (1.0–2.5)	19%	1.3 (0.8–2.2)	3%	13.7 (9.4–21.5)	46%	2.4 (1.6–3.8)	24%	9.6 (6.3–16.2)	21%
PHC-focused	Programme implementation costs (average of 2022–2026)	6.3 (5.9–6.6)	18%	13.1 (12.6–13.5)	164%	18.1 (17.6–18.7)	39%	11.9 (10.3–13.8)	40%	2.8 (2.5–3.1)	28%	26.9 (25.4–28.6)	60%
	Routine costs (2026)	0.7 (0.4–1.0)	2%	0.5 (0.4–0.8)	6%	0.4 (0.3–0.6)	1%	5.1 (3.5–7.3)	17%	0.8 (0.6–1.2)	8%	3.4 (2.3–4.9)	8%

OPD, patients presenting at outpatient department; DH, district hospital; PHC, primary health centre; SOC, standard of care; NTP, National Tuberculosis Programme.

Costs are in 2021 US$.

The average annual cost of a five-year scale-up plan of the DH-focused intervention ranged between $3 M (Cambodia) and $10 M (Mozambique), corresponding to between 8% (Cambodia) and 54% (Cameroon) of countries' national tuberculosis programme (NTP) budgets in 2021, and, for the PHC-focused intervention, between $3 M (Sierra Leone) and $27 M (Uganda), or 18% (Cambodia) to 164% (Cameroon) of NTP budget. Once the health facilities are equipped, the average annual routine costs (corresponding to year 2026) ranged between $1.3 M (3% NTP budget) in Côte d’Ivoire and $13.7 M (46%) in Mozambique for DH-focused, and between $0.4 M (1%) in Côte d’Ivoire and $5.1 M (17%) in Mozambique for the PHC-focused strategy.

We assumed facilities saw a sustained increase in diagnoses following implementation. We projected an increase in the cumulative number treated for tuberculosis over the five-year implementation period of 194% for the DH-focused, and 24% for the PHC-focused strategy, compared to SOC. Sensitivity analyses suggested that important cost reductions could be achieved through purchase price reduction of the cartridge kits and mucus aspirators, and economies of scale on delivery chain costs (Appendix section V).

## Discussion

Benchmarked against estimates of country cost-effectiveness thresholds,^18^ this study found that the decentralisation of paediatric tuberculosis services to DH level could be cost-effective compared to the SOC from a health systems perspective in Cambodia and Côte d’Ivoire, whereas decentralisation to PHC level was unlikely to be cost-effective in any country. Ultimately, choice of cost-effectiveness thresholds is a judgement for decision-makers, and other considerations, notably the budget impact, also need to be considered. Sensitivity analysis suggests that decentralisation targeted to geographical areas with very high tuberculosis prevalence would be highly likely to be cost-effective in all countries. Implementation would require substantial financial investment in the early phase, particularly for the PHC-focused intervention.

The PHC-focused strategy was, as expected, more costly than the DH-focused strategy due to spending on diagnostic equipment for a larger number of facilities, but unexpectedly was also less effective than the DH-focused strategy. The reasons for this lower effectiveness (fewer tuberculosis diagnoses) are not clear, but may include differences in staff cadres composition and greater experience in diagnosing paediatric tuberculosis at DH level due to higher patient volumes.^12^ The higher tuberculosis diagnosis rate in children seeking care at DH level may indicate care-givers prefer to take more severely ill children to this level first or because children are referred from PHC with more advanced disease. We did not model differences in tuberculosis severity by location which may have favoured the DH-focused strategy. Finally, all children enrolled in DH had a CXR performed while in PHCs, only children with persisting symptoms after 7 days were referred for CXR at DH; this is likely to have contributed to higher tuberculosis detection in the DH-focused strategy. We did not include patient costs in our analysis; the PHC-focused strategy is likely to have reduced costs to patients due to fewer referrals.

Our study has some limitations. Given the study design and following our pre-specified analysis plan, we did not use country-specific estimates of health impact and only accounted for country-specific variation on the cost side. We used a single typical SOC as a comparator across the six countries. It was not practical or transparent to represent every variation that exists within and across these countries. In our iterative conceptual modelling, we agreed on a structure clinicians from all countries felt adequately represented patient pathways in their country. However, paediatric tuberculosis care was already partially decentralised to PHC level in Uganda and Mozambique, meaning our modelled SOC is more of an approximation in these settings. Some parameters were derived by calibrating to cascade data, and our estimate of the true tuberculosis prevalence in children seeking care across all countries was uncertain. In reality tuberculosis prevalence will vary between countries, and higher prevalence values substantially improved cost-effectiveness (Appendix section IV).

We were also limited by the nature of the TB-Speed Decentralization study and data. The pre−/post-intervention primary outcome means results are vulnerable to confounding by factors that changed over time. In particular, the study period overlapped with the COVID-19 pandemic. Restrictions introduced by countries affected transportation (less available and more costly) and these barriers may have increased patient losses during referral to hospitals. Fear of COVID-19 also reduced facility attendance. Costs were sourced from national pay scales, project accounts, the Stop TB partnership Global Drug Facility catalogue or from the WHO-CHOICE database, whereas laboratory resources used were shared by laboratory managers of the study sites (located in rural or semi-urban districts), therefore, costs might not always be representative of the whole country. Facility-level overheads costs were recently reported to be a significant contributor to total costs in adult tuberculosis costing estimates from the Value-TB project (the largest tuberculosis costing effort to date).^22^ These were excluded in our study because it was anticipated that the adoption of a new diagnostic strategy without change to the number of health facilities or staff would not significantly affect overheads, but this assumption should be carefully considered alongside roll-out plans. We also neglected the contribution to morbidity to DALYs, but this has been shown to be an excellent approximation.^23^ Data collection, as well as our analysis, was focused on children, which ignores the additional benefits from decentralisation of tuberculosis diagnostic capacity in improving the detection of tuberculosis in adults, as well as detection of rifampicin-resistant tuberculosis and other pathogens using the GeneXpert platform.

A major strength of this study is the collection and analysis of primary data to inform costs, impacts, and care cascades in six high tuberculosis incidence countries in different regions of Africa and in South-East Asia. This diversity in economic and health system context suggests the generalisability of these results across comparable settings. Underlying modelling assumptions were based on a number of previously published studies, and applied within a framework that captured considerable complexity in patient pathways, including reassessments and referrals between levels.

To our knowledge, this is the first multi-country study to assess the cost-effectiveness or budget impact of decentralising childhood tuberculosis services, and will add to the evidence base for the interim WHO recommendation on decentralised models of care. Systematic reviews of the cost and cost-effectiveness of tuberculosis screening have found ICERs of between US$281 and US$698 per DALY averted in the general population,^24^ but for screening in high risk groups ICERs as low as US$51 per DALY have been reported.^25^ An analysis of the cost-effectiveness of Xpert on stool for paediatric tuberculosis in Ethiopia and Indonesia found ICERs of US$132 and US$94 per DALY, respectively.^26^ However, the majority of these analyses were not based on empirical data from implemented interventions, and restricted to a small component of tuberculosis diagnosis given that the majority of children are clinically diagnosed.

A number of studies have explored the effects of decentralisation of general tuberculosis services and costs. A 2017 study assessed the cost per tuberculosis diagnosis of implementing Xpert testing regardless of age at PHCs in Uganda.^27^ Average costs per new tuberculosis diagnosis using Xpert averaged US$119, but were as high as US$885 in the lowest-volume centre. The authors did not attempt to estimate cost-effectiveness in terms of cost per DALY averted. Thompson and colleagues estimated the cost-effectiveness of an adult tuberculosis diagnostic strategy decentralised in community health centres in Uganda.^28^ The authors recommended decentralised testing services with ICERs ranging US$687 per additional treatment initiation in 14 days and US$1332 per additional tuberculosis diagnosis. Cost-effectiveness notably increased with high testing volumes and lower equipment costs.

TB-Speed Decentralization and other studies have demonstrated the potential for decentralised diagnostic approaches to find more children with tuberculosis.^12^^,^^29^ Our analysis shows that this is possible in a way that could be considered cost-effective across a range of settings, depending on strategy and tuberculosis prevalence, but that large-scale implementation would still incur substantial costs relative to existing NTP budgets. Countries should consider scaling up locally-adapted interventions to improve tuberculosis diagnosis while monitoring their performance, potentially prioritising areas with the highest tuberculosis prevalence, and including such plans when identifying domestic or donor sources of funding.

## Contributors

MH, NM, PD, OM, MB, EW conceived the health economics analysis plan. PD developed the cost-effectiveness model and MDE developed the budget impact model. MDE and NM had access to and verified the underlying study data. MDE conducted the cost, the cost-effectiveness and the budget impact analyses. MH coordinated international economic data collection and design of the patient pathways. MDE, PD, MH, NM interpreted economic results. TB, AB, HS provided scientific expertise and guidance.

OM, MB, EW conceived and designed the TB-Speed Decentralization study, and acquired the project financial support. MN coordinated and contributed to the implementation of the study protocol. OM, MB, and EW led the study at international level. LB led the study in Cambodia, J-VT led the study in Cameroon, RM led the study in Côte d’Ivoire, CK led the study in Mozambique, JM led the study in Sierra Leone, EW and JM-A led the study in Uganda, MN coordinated study implementation at international level. MHTNN did the statistical analysis of the decentralisation study. OM, MN, MH, MB, EW and MDE contributed to the interpretation of the results.

MDE wrote the first draft and all authors reviewed, edited and approved the final version of the manuscript. MDE, PD, OM, MB, EW were responsible for the decision to submit the manuscript.

## Data sharing statement

Aggregated data for all analyses will be publicly available with the publication on a GitHub repository under a Creative Commons Attribution (CC BY) licence (URL: https://github.com/petedodd/TBSdecent).

## Declaration of interests

MH was paid as a subcontractor by the University of Bordeaux from the Unitaid grant for the TB-Speed programme. MB is employed by the Institut de Recherche pour le Développement (TransVIHMI) who received Unitaid funds in relation to this study. MB is the chair of the board of Epicentre since November 9th, 2022, which was a third party in the TB-Speed project who received funds from Unitaid, but MB did not receive any payment for this activity. PD is subcontracted on the Unitaid grant through the partnership between the University of Bordeaux and the University of Sheffield. All other authors declare no competing interests.

## Appendix A Supplementary data

Appendix TB-Speed Decentralization

Pubmed TB-Speed Health Economics Study Group
