# Supplementary material for: Cost-effectiveness and budget impact of decentralising childhood tuberculosis diagnosis in six high tuberculosis incidence countries: a mathematical modelling study
Source: eClinicalMedicine. 2024 Mar 21;70:102528. doi: 10.1016/j.eclinm.2024.102528 (PMC11056392; doi:10.1016/j.eclinm.2024.102528)
Supplement: Pubmed TB-Speed Health Economics Study Group [file mmc2.docx]

| **First name** | **Surname** |
| --- | --- |
| Doris | Arlt-Hilares |
| Eric | Balestre |
| Marie-France | Banga |
| Antoine | Bénard |
| Tanguy | Bernard |
| Maryline | Bonnet |
| Laurence | Borand |
| Guillaume | Breton |
| Dim | Bunnet |
| Paul-Damien | Chateau |
| Saniata | Cumbe |
| Marc | d’Elbée |
| Agathe | de Lauzanne |
| Peter, James | Dodd |
| Martin | Harker |
| Minh | Huyen Ton Nu Nguyet |
| Sanary | Kaing |
| Celso | Khosa |
| Eric | Komena |
| Monica | Koroma |
| Sylvie | Kwedi Nolna |
| Nyashadzaishe | Mafirakureva |
| Tan Eang | Mao |
| Olivier | Marcy |
| Douglas | Mbang Masson |
| Raoul | Moh |
| Jacob | Mugisha |
| Ayeshatu | Mustapha |
| Juliet | Mwanga-Amumpere |
| Mastula | Nanfuka |
| Naome | Natukunda |
| Joanna | Orne-Gliemann |
| Eric | Ouattara |
| Julien | Poublan |
| Hojoon | Sohn |
| Jean-Voisin | Taguebue |
| Immaculate | Tulinawe |
| Yara | Voss de Lima |
| Jérôme | Wittwer |
| Eric | Wobudeya |
